# Healthy Live Births after the Transfer of Mosaic Embryos: Self-Correction or PGT-A Overestimation?

**DOI:** 10.3390/genes15010018

**Published:** 2023-12-21

**Authors:** Gerard Campos, Romualdo Sciorio, Steven Fleming

**Affiliations:** 1 Geisinger Medical Center, Women’s Health Fertility Clinic, Danville, PA 17821, USA; gerardcampos10@gmail.com; 2 GIREXX Fertility Clinics, C. de Cartagena, 258, 08025 Girona, Spain; 3 Fertility Medicine and Gynaecological Endocrinology Unit, Department Woman-Mother-Child, Lausanne University Hospital, 1011 Lausanne, Switzerland; 4 Discipline of Anatomy & Histology, School of Medical Sciences, University of Sydney, Sydney, NSW 2006, Australia; blueyfleming@gmail.com

**Keywords:** mosaicism, trophectoderm biopsy, rebiopsy, preimplantation genetic testing, self-correction, overestimation, intermediate copy number

## Abstract

The implementation of next generation sequencing (NGS) in preimplantation genetic testing for aneuploidy (PGT-A) has led to a higher prevalence of mosaic diagnosis within the trophectoderm (TE) sample. Regardless, mosaicism could potentially increase the rate of live-born children with chromosomic syndromes, though available data from the transfer of embryos with putative PGT-A mosaicism are scarce but reassuring. Even with lower implantation and higher miscarriage rates, mosaic embryos can develop into healthy live births. Therefore, this urges an explanation for the disappearance of aneuploid cells throughout development, to provide guidance in the management of mosaicism in clinical practice. Technical overestimation of mosaicism, together with some sort of “self-correction” mechanisms during the early post-implantation stages, emerged as potential explanations. Unlike the animal model, in which the elimination of genetically abnormal cells from the future fetal lineage has been demonstrated, in human embryos this capability remains unverified even though the germ layer displays an aneuploidy-induced cell death lineage preference with higher rates of apoptosis in the inner cell mass (ICM) than in the TE cells. Moreover, the reported differential dynamics of cell proliferation and apoptosis between euploid, mosaic, and aneuploid embryos, together with pro-apoptosis gene products (cfDNA and mRNA) and extracellular vesicles identified in the blastocoel fluid, may support the hypothesis of apoptosis as a mechanism to purge the preimplantation embryo of aneuploid cells. Alternative hypotheses, like correction of aneuploidy by extrusion of a trisomy chromosome or by monosomic chromosome duplication, are even, though they represent an extremely rare phenomenon. On the other hand, the technical limitations of PGT-A analysis may lead to inaccuracy in embryo diagnoses, identifying as “mosaic” those embryos that are uniformly euploid or aneuploid. NGS assumption of “intermediate copy number profiles” as evidence of a mixture of euploid and aneuploid cells in a single biopsy has been reported to be poorly predictive in cases of mosaicism diagnosis. Additionally, the concordance found between the TE and the ICM in cases of TE biopsies displaying mosaicism is lower than expected, and it correlates differently depending on the type (whole chromosome versus segmental) and the level of mosaicism reported. Thus, in cases of low-/medium-level mosaicism (<50%), aneuploid cells would rarely involve the ICM and other regions. However, in high-level mosaics (≥50%), abnormal cells in the ICM should display higher prevalence, revealing more uniform aneuploidy in most embryos, representing a technical variation in the uniform aneuploidy range, and therefore might impair the live birth rate.

## 1. Introduction

Aneuploidy or abnormal chromosome copy number is clearly associated with pregnancy failure, and most abnormalities are not viable, fail to implant, or are lost later in pregnancy [1,2,3]. While chromosome segregation errors that occur during meiosis lead to aneuploidies present uniformly across the embryo, those produced after fertilization during mitotic divisions originate from non-disjunction, anaphase lag, or chromosome breaks, resulting in mosaicism for both whole and partial chromosomes [4,5]. Current preimplantation genetic testing for aneuploidy (PGT-A) combined with trophectoderm (TE) biopsy is based on molecular techniques that analyze the DNA of 5–10 biopsied cells, including array comparative genomic hybridization (aCGH), single-nucleotide polymorphism (SNP) array, quantitative polymerase chain reaction (qPCR), and, more recently, next-generation sequencing (NGS), which has become the prevalent option [6]. At the blastocyst stage, the NGS approach not only allows viable embryos to be selected but is also able to identify full aneuploidies, and thus, results of intermediate copy number leading to a mosaicism diagnosis are common [7]. The incidence of mosaicism mostly ranges between 5 and 15% [7,8,9], even though higher variability has been reported between clinics [10]. Inter-clinic variation may be related to different testing laboratory practices, the predominant patient age group involved, stimulation protocols and embryo culture conditions, such as an increase in oxidative stress that may lead to segregation errors [11,12]. Most mitotic failures are thought to arise during the first cell divisions when embryo development is highly dependent on maternal mRNA and proteins [13]. Cell cycle control during the transition from maternal to embryonic gene expression is suggested to be laxer; therefore, in the event of chromosome misalignment, cell cycle checkpoints may fail to pause the cell progression to resolve the disequilibrium [14,15]. The dysregulation of mitosis in early embryos would make them particularly vulnerable to segregation errors and mosaicism [16]. An early segregation error would presumably lead to a mosaic embryo with a higher percentage of aneuploid cells [17]. Although aneuploid embryos are routinely discarded due to an increased risk of miscarriage and congenital disorders in newborns [3], the clinical management of mosaic embryos with both aneuploid and euploid linkages remains a subject of debate. Evidence suggests that many embryos classified as a mosaic by PGT-A can result in healthy births, though with increased implantation failure and miscarriage rates [7,18,19,20,21,22]. Indeed, mosaic embryo outcomes seem to be related to the type of aneuploidy involved (segmental, or involving one or two chromosomes, and complex aneuploidies with three or more chromosomes affected) and the percentage of aneuploid cells in the embryo [20,21,22]. Although the Preimplantation Genetic Diagnosis International Society position is against the use of a fixed cut-off value for their transfer management, according to the available data, “low-level mosaics” (<50% aneuploid cells in the embryo) seem to have better outcomes compared to embryos with a “high-level” of mosaicism, showing a comparable reproductive potential with uniformly euploid embryos [10,19,20,21,22,23]. The main concern is whether the transfer of mosaic embryos could result in a higher frequency of congenital abnormalities affecting future offspring health. Since Greco and co-authors [24] reported, for the first time, a healthy pregnancy after replacing mosaic embryos and demonstrated their developmental potential, nearly all studies have revealed healthy pregnancies and births when embryos deemed as mosaic were transferred. To date, there is no evidence correlating mosaicism to a higher risk of negative fetal or neonatal outcome. Only rarely, a fetal aneuploidy originating from a mosaic embryo (for the same chromosome) has been confirmed with advanced prenatal or postnatal stages. One case resulted in a non-syndromic, phenotypically healthy baby with 2% mosaicism in one tissue after the transfer of a “low level” (35%) mosaicism embryo [25]. The other has recently been reported as a liveborn with partial trisomy 15 and maternal uniparental disomy (UPD) 15 in a likely non-mosaic form as a result of a double embryo transfer involving a “high-level” mosaic embryo for trisomy 15 and a deletion in the long arm of chromosome 20 [26]. Recently, a segmental duplication (+4q32.2q34.3) conserved from the embryonic stage led to an apparently healthy newborn with no gross birth abnormalities [20]. The only case entailing a major congenital anomaly (as considered by the Centers for Disease Control and Prevention) involved a fetal non-mosaic duplication with a similar chromosomal location to a mosaic duplication diagnosed in the preimplantation embryo. An altered phenotype including a coarctation of the aorta was reported in the newborn [20]. The same group gathered prenatal testing data from 250 pregnancies to determine the persistence of the embryonic mosaicism identified with PGT-A throughout the pregnancy. Data from the analysis of eight products of conception from miscarried or aborted pregnancies were also included. Apart from the one that resulted in the heart’s major congenital anomaly, two additional cases reported that preimplantation mosaicism persisted through the pregnancy. The PGT-A diagnosis of low-level mosaicism was reconfirmed by amniocentesis in both cases, and thus the patients opted to terminate the pregnancies. In summary, the persistence of mosaicism during gestation was 1.2% (3/250) [20]. These results are consistent with prenatal diagnosis data found among the general population, in which 1–2% of chorionic villi samples are diagnosed as a mosaic [27,28]. Considering that no increased evidence of aneuploidy or congenital abnormalities has been reported, not only after replacing PGT-A mosaics but also following thousands of mosaics and aneuploid embryos “blindly” being transferred in those cycles without genetic testing [11], several hypotheses have been postulated to explain the disappearance of the aneuploid cell population at birth. There are two main possible explanations: (1) the embryo is able to self-correct its chromosome complements during its development or (2) the PGT-A diagnosis does not reflect the embryo’s karyotype, because either the TE status is not representative of the whole embryo or there is an overestimation of embryo diagnoses as mosaic (false positives).

## 2. Self-Correction and Embryo Plasticity

Beyond standard and well-defined embryo development, signs of embryo plasticity are increasingly evident. Although several perturbations can affect embryo viability and implantation potential, they are still proven to be compatible with implantation and live-term pregnancies. The ability to reverse binucleation at the two-cell stage in the next cell division, which potentially might alter cell compaction, the morula stage and blastocyst [29,30,31,32], and the capacity to overcome cell loss from cryopreservation and develop to term, are examples of human embryo plasticity. Several studies have reported decreasing aneuploidy rates in preimplantation embryos, suggesting their capacity to self-correct and normalize their chromosomal content. Munnè and colleagues [33] found a chromosome normalization in 23 aneuploid embryos previously diagnosed by fluorescence in situ hybridization (FISH) at day 3, as having an increase in euploid cell rate from day 6 (average of 13%) to day 12 (average of 48%). Subsequently, 7 embryos became euploid and 11 were identified as mosaic, with between 21% and 88% of normal cells. A further study based on FISH data also showed a reduction in aneuploid cells in 32.6% of the embryos diagnosed as aneuploid and mosaic at day 3 when they were reanalyzed on day 5, reporting a total normalization in 9.7% of the 83 embryos included [34]. Extended in vitro embryo culture and the introduction of NGS corroborated these findings when 71% of the embryos originally diagnosed as a mosaic at day 5 were reported to be euploid at day 12 and, following extended culture, normal profiles were identified not only in the TE-derived lineages but also in the inner cell mass (ICM) [35].

## 3. The Mortality Model (Apoptosis and Depletion)

**Figure 1:**
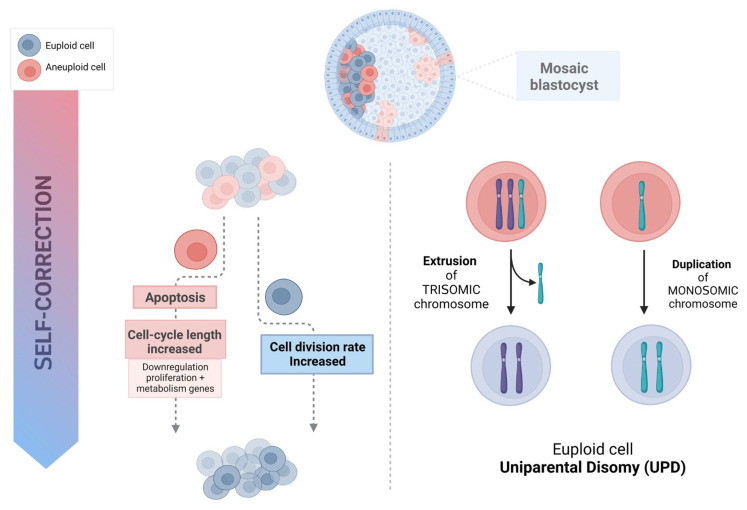
Possible mechanism of human embryo self-correction.

Aneuploid cells may be eliminated and progressively depleted from a mosaic embryo through selective apoptosis and reduced proliferation, with slower cell cycles of the abnormal cells [32]. This self-correcting ability to eliminate genetically abnormal cells from the future fetal lineage has been demonstrated in mice [36]. To investigate the fate of aneuploid cells during pre- and post-implantation development, a mouse model of euploid–aneuploid mosaicism was generated using the drug Reversine, an inhibitor of monopolar spindle 1-like 1 kinase [37], which inactivated the spindle assembly checkpoint and induced high rates of chromosomal segregation errors. This study reported a significant decrease in the percentage of altered blastomeres in each mosaic (1:1 Reversine-treated and control blastomeres) embryo from the early-stage to late-stage blastocyst (53% to 47%, *p* < 0.01), mainly due to a reduction of abnormal cells from 55.8% to 44.2% (*p* < 0.05) in the ICM but not a significant reduction in the TE. The preferential allocation of abnormal blastomeres to different cell lineages was tracked using high-resolution time-lapse imaging. Importantly, not only was apoptosis reported to be present in most mosaic embryos at the blastocyst stage, with apoptotic features detected in 30.9% of the ICM cells and fewer (2%) in the TE, but also the frequency of apoptosis was significantly higher in the aneuploid group of cells than in the euploid group, both in the ICM (41.4% vs. 19.5%, *p* < 0.001) and the TE (3.3% vs. 0.6%, *p* < 0.001). Hence, apoptosis proved to be a mechanism to eliminate chromosomally abnormal cells in mice, especially in the ICM, while TE cells were progressively depleted because of increased cell cycle length and senescence. Cell cycle lengths were significantly longer in TE aneuploid cells (not in ICM abnormal cells) than in normal cells (9.2% of the TE abnormal slower cells versus 1.4% of the TE cells from the control, *p* < 0.001). Progressive depletion of aneuploid cells in the preimplantation embryo was established through different mechanisms related to specific cell lineages, with apoptosis as the primarily responsible means in the ICM, while in the TE, the aneuploid cells would be negatively selected and reduced in relation to their euploid counterparts due to their longer cell cycles. This asynchrony in eliminating abnormal cells could explain the differences between the ICM and TE, especially when high rates of mosaicism are present. Even though it has been demonstrated in animal models, differences in cell cycle regulation and the timing of development between mice and humans [38], together with the limitations regarding the methodology and reagents permitted for human use, make it difficult to confirm this hypothesis in human embryos. However, the use of human “grastruloids”, created from RUES2 (NIHhESC-09-0013) human embryonic stem cells treated with Reversine, recently provided a human model for aneuploid cell fate in preimplantation human development [39]. A significant enhancement of apoptosis in the embryonic germ layer displayed an aneuploidy-induced cell death lineage preference, showing that pluripotent epiblast and TE cells were remarkably resilient to aneuploidy with significantly lower levels of apoptosis. These findings support a similar underlying mechanism in humans [39]. Furthermore, in human blastocysts, Victor and colleagues [40], using immunofluorescent markers of mitosis (Phosphohistone 3) and apoptosis (Caspase-3), were able to report differential dynamics of cell proliferation and death between euploid, mosaic, and aneuploid embryos. Abnormal blastocysts showed significantly higher rates of apoptosis (ICM, *p* < 0.05; TE, *p* < 0.001) and increased rates of cell division (ICM, *p* < 0.05; TE, *p* < 0.01), presumably compensating for the slower proliferation of aneuploid cells or their loss by programmed cell death [40]. An alternative approach based on gene expression profiles of embryos from day 4 morulae to day 7 blastocysts showed upregulated immune response genes and, more importantly, the downregulation of genes involved in proliferation and metabolism in the aneuploid cells of mosaic embryos [41]. Data from human blastocoel fluid (BF) support selective apoptosis as a self-correction mechanism that may rescue embryos from aneuploidy (Figure 1) [42]. The presence of cell-free DNA (cfDNA) and other molecular remnants in BF may suggest an apoptotic origin during early embryonic development. Though the biological mechanisms by which embryonic DNA from lysed or partially lysed cells is released into the blastocoel are difficult to define, it may potentially originate from cells undergoing apoptosis [43,44]. Testing the blastocoel cfDNA of euploid blastocysts developed from embryos previously diagnosed as aneuploid on day 3, Tobler and collaborators [45] found that 86% (12 of 14) of normalized embryos on day 5 still showed aneuploid results within the BF, suggesting that abnormal cells may be marginalized during blastulation. Recent data support the extrusion of this aneuploid embryonic DNA; specifically, when several structures of the blastocyst derived from day 3 aneuploid embryos were analyzed, the rate of aneuploidy was found to be significantly higher (*p* < 0.0001) in the BF (78%) than in the ICM (39%) and TE (49%), with nearly all abnormalities concordant with day 3 diagnosis [46]. Blastocoel fluid has been reported to contain cfDNA as well as mRNAs encoding apoptotic genes [47,48]. The identification of these pro-apoptotic gene products together with extracellular vesicles also found within the BF, provides additional support for the hypothesis of apoptosis as a mechanism that purges the preimplantation embryo of aneuploid cells [49].

## 4. Trisomy/Monosomy Rescue Model

Correction of aneuploidy by extruding a trisomy chromosome or by monosomic chromosome duplication could theoretically represent a mechanism to explain success after the transfer of “mosaic” embryos [32,50], leading to UPD. In such a case, two copies of the chromosome are inherited from the same parent with no representative copy from the other; therefore, if imprinted genes or harmful recessive alleles are involved, this may result in syndromic newborns. Nevertheless, the low prevalence of UPD (from 0% to 0.06%) found in human embryos [50,51] suggests that, though possible, it is an extremely rare phenomenon. These results were recently corroborated by data from the general population, where the UPD rate was estimated to be 1 in 2000 euploid, liveborn individuals (rate: 0.05%; 99% CI: [0.04–0.06%]) [52], which would hardly explain the rescue of mosaic embryos into healthy euploid babies. Moreover, Scuffins and co-workers [53] reported the presence of UPD involving imprinted chromosomes in only one out of 320 syndromic infants born.

## 5. Misinterpretations of PGT-A Results

### 5.1. Technical Accuracy

The technical limitations of PGT-A may lead to inaccuracy in embryo diagnoses, identifying as “mosaic”, embryos that are in fact uniformly euploid or aneuploid [40,54,55]. PGT-A using NGS is usually performed as a method of quantifying chromosomes to profile the karyotype following TE biopsy, using chromosome copy number thresholds to predict the euploid, aneuploid, or mosaic status of the embryo. Briefly, 24-chromosome copy number analysis by NGS involves fragmenting the whole-genome amplified DNA sample into hundreds of thousands of small fragments (100–200 base pairs) that are sequenced in parallel. Sequencing of each fragment, which requires the addition of fluorescent nucleotides and ultrahigh-resolution imaging technology, continues until a sufficient “read depth” (the number of sequence reads for the same genomic region) is acquired. Then, these sequences are compared with the reference genome and counted using specific software. As a result, a specific number of reads from a given chromosome is proportional to the copy number in case of euploidy, while greater or lower read depth would entail trisomy or monosomy, respectively. Far from being analyzed and karyotyped individually, NGS collectively analyses the amount of DNA for each chromosome from a group of cells (multicellular TE biopsy) using a bioinformatics algorithm to compare it with a normal copy number reference value. The NGS approach requires whole genome amplification (WGA) followed by a library construction, sequencing, and alignment of readings with the human reference genome. More precisely, this necessary DNA extraction and amplification, known to be susceptible to errors, together with other methodological issues (i.e., undetected sample contamination, suboptimal polyploidy, or the bioinformatic algorithms used) may affect the level of noise observed, which may result in artifactual intermediate copy numbers and contribute to an overestimation of chromosomal mosaicism in clinical practice [35,56,57,58]. In addition to the DNA standard amplification technologies’ propensity for errors, the high variability of TE biopsies, in terms of quantity and quality (intact cells together with fragmented cellular remnants) may lead to intermediate copy number results and therefore to false-positive mosaicism profiles. An insufficient or excessive number of cells may have a significant impact on the PCR amplification plot at the time of quantification, which could result in an underestimate of the relative amount of DNA [23].

Different genetic testing laboratory practices (i.e., cut-off values used) may entail different levels of accuracy (sensitivity and specificity) and may therefore have significant impact upon the mosaicism rate reported [54]. Most validation studies are based on models employing mixtures of euploid and aneuploid cell lines at different ratios that intend to mimic the variation found in in vivo samples. These cell mixes have been mostly developed from the genomic DNA of cell lines with different well-defined chromosome complements [59] or, alternatively, by merging well-defined proportions of euploid and aneuploid cells [60]. However, these models represent a highly stable scenario, with no variation in the quantity or quality of cells analyzed, which certainly differs from blastocyst biopsy specimens, characterized by an uncertain number of intact cells together with fragmented cellular remnants derived from technical procedures. Consequently, any extrapolation of mosaicism from these idealistically stable models might constrain the diagnostic efficiency of NGS in diagnosing it [54].

The cut-off values represent analytical noise levels and specific technological variations [18]. Some laboratories employ more dynamic ranges (i.e., 20–80% thresholds), in which chromosome copy number deviations less than 20% (1.8≥ and ≤2.2) are reported as euploidy and greater than 80% as aneuploid (i.e., 0.8≥ and ≤1.2 for aneuploidy; 2.8≥ and ≤3.2 for triploidy) while others accept more conservative cut-off values, i.e., from 30% to 70%, and consequently higher analytical noise levels [10,56]. Data analysis may reveal intermediate copy numbers outside these ranges for the two normal copies and full monosomies or trisomies. These results are presumably consistent with the presence of both euploid and aneuploid cells among the biopsied TE and are therefore profiled as mosaics [57]. However, different factors may affect the level of noise observed (i.e., undetected sample contamination, suboptimal DNA amplification, polyploidy, or the bioinformatic algorithms used), which may result in artifactual intermediate copy numbers and contribute to an overestimation of chromosomal mosaicism in clinical practice [35,57,58]. Nevertheless, data from one systematic review involving reanalysis of embryos deemed mosaic demonstrated a high discordance when mosaicism was diagnosed using NGS testing based upon intermediate copy numbers [10]. Embryo reanalysis included TE rebiopsies, ICM sampling, whole embryo screening and blastocyst outgrowths analysis. The accuracy of embryo diagnosis was reported to be lower with NGS, displaying an euploidy concordance of 92.2% compared to 97.1% (*p* = 0.0053) when NGS was not used in the original biopsy, and showed even less concordance in cases with a full aneuploidy diagnosis: 75.9% with NGS and 94.8% without NGS (*p* < 0.0001). Particularly poor was the predictive value for the embryos placed in the mosaic range by NGS, as the concordance of mosaic aneuploidy with the remaining embryo was only 42.6%. Consequently, these data suggested that even if NGS technology accurately detects euploidy, it is significantly inaccurate for aneuploidy, and highly inefficient in diagnosing mosaicism. Moreover, the reanalysis of the mosaic embryos revealed that most of them (57.4%) were unlikely to present mosaicism, reporting euploidy in 29% of embryos and 28.4% of full aneuploids. This lack of specificity of mosaicism predictions was corroborated recently by Handyside and co-authors [57], who performed SNP genotyping and karyomapping to follow-up embryos identified by NGS-based PGT-A as mosaic. In addition to detecting overestimation of mosaicism, they were able to identify a significant proportion of embryos with meiotic aneuploidies. Only 1 out of 21 (4.8%) cases diagnosed as putative mosaic was confirmed, 42.8% were euploid, and 47.6% were found to be aneuploid, including embryos with meiotic trisomies, monosomies, and triploidies [57]. The significant discordance upon reanalysis would lead to the consideration of false-positive mosaic classification as an alternative hypothesis for the clinical outcomes found after transferring embryos diagnosed as putative “mosaic” by NGS and chromosome copy number analysis. Therefore, uniform aneuploid embryos misdiagnosed as mosaic would lead to negative reproductive consequences [3], while the healthy deliveries reported would be the result of transferring truly euploid embryos misdiagnosed as mosaic [10,23].

### 5.2. Concordance between TE and ICM

Estimates based on a small biopsy (5–10 cells) could not possibly be representative of the whole embryo if aneuploid cells are not distributed evenly. Biologically, the moment when mosaicism arises influences the location and distribution of aneuploid cells. Importantly, when performing PGT-A, the biopsy takes a few cells from the TE, the precursor to the placenta, but not from the ICM, the cell lineage from which the fetus arises. Thus, it remains an indirect estimation of the entire embryo’s status and does not prove the surrounding TE cells’ karyotype. Indeed, at least theoretically, according to mathematical modelling, a single TE biopsy could not reliably determine the genetic status of the remaining embryo, so its clinical utility would be questionable [61]. Data on the distribution of aneuploid cells within mosaic embryos are scarce; most studies have reported a poor concordance between the TE and the rest of the embryo [7,55,62,63], while others have found that the TE karyotype is a relatively accurate predictor of ICM chromosomal status [40,64,65,66]. In one study, five blastocysts initially classified as mosaics were re-analyzed using ICM biopsy and one additional sample from the TE [55]. Even with the limited sample size, they identified as euploid the ICM of 3/5 blastocysts and the subsequent TE biopsy in 2/5 embryos, demonstrating that despite being classified as mosaic, the embryo can be euploid in other regions and that clinically diagnosed mosaicism from a single biopsy is not a good predictor of the whole embryo’s karyotype. Nevertheless, the same group reported 96.8% (n = 93) of clinical TE-ICM concordance in blastocysts classified as “uniform aneuploids”, highlighting that this experimental evidence does not apply to mosaic diagnoses [40]. Similarly, Huang and colleagues [66] analyzed 51 donated abnormal blastocysts and reported TE aneuploidy as an outstanding predictor of ICM imbalance but showed a much lower correlation for mosaicism. The ICM and three different separated TE regions (opposite, upper right, and lower right of the ICM) were biopsied and analyzed by aCGH, reporting 84.3% of embryos with consistent results in all four biopsies. Interestingly, when discordance was identified between one of the TE regions and the rest of the samples (mosaicism), these aneuploid cells had no special location among the areas biopsied, being not limited to a specific region of the TE. A similar result was reported after reanalysis via FISH of previous genetic diagnosis obtained by TE aCGH analysis, showing no preferential allocation of abnormal cells in euploid/aneuploid mosaics, which were evenly distributed across the blastocyst [65,67]. In contrast, recent conclusions from a dataset of disaggregated human blastocysts are distinctly noteworthy [7]. The prevalence and distribution of aneuploid cells was studied by NGS in 91 blastocysts, from which the ICM was isolated, and the TE was divided into four pieces, with the ICM labelled separately. When the concordance between the reference TE and the rest of the portions was analyzed, they found significant differences depending on the chromosomal content of the baseline TE biopsy. Interestingly, when high level mosaicism (50–70%) was reported in the diagnostic TE sample, 65% of blastocysts were uniformly aneuploid in the ICM and the rest of the analyzed pieces. However, when aneuploid cells represented less than 50% in the TE biopsy, meaning they had been classified as euploid or low-medium level mosaics, chromosomal abnormalities were extremely rare (1% of cases) across the ICM or affected other portions of the embryo. Indeed, the distribution of aneuploid cells and their impact on the embryo were equivalent between euploid and low-medium level mosaics (*p* = 0.14). They concluded that low-medium level mosaicism diagnoses would be consistent with postzygotic errors in chromosome segregation that emerge after TE and ICM differentiation, which would confine aneuploidy to a specific area rather than evenly across the whole blastocyst [7]. Consistent with these results, true incidence of mosaicism was also confirmed by Wu and colleagues [63] when they reanalyzed 101 mosaic embryos and found a concordance with the ICM of only 27.5%, while high rates of euploidy (63.7%) and low rates of aneuploidy (8.8%) were disclosed. Additionally, increased rates of full aneuploidy (≥37.5%) were reported after the rebiopsy of high-level mosaics (≥60%) compared with the 2.6% of true aneuploids found when low-level mosaicism was initially diagnosed. This study also revealed differences related to the type of mosaicism, displaying a lower ICM concordance for segmental-chromosome mosaicism than for results implying whole chromosomes [63]. More recently, another study has shed additional light on the competence of the TE biopsy to reflect any part of the remaining embryo [68], after splitting the entire embryo into four pieces and analyzing their genetic content. An extremely low confirmation rate in all the rebiopsies was reported for mosaicism, both for whole-chromosome (2.29%) and segmental aneuploidy (2.15%), while the partial embryo concordance (diagnoses confirmed in at least one sample) were 39.08% and 41.94%, respectively (Table 1). By extension, a scattered but not uniform distribution of mosaicism was inferred by the authors, although it was a general model because they did not distinguish different patterns between low, medium, and high levels of mosaicism.

## 6. Discussion

The implementation of NGS in PGT-A allows accurate detection of aneuploidies but it has significantly increased prevalence of mosaic diagnosis. Even though the data on the outcomes after a mosaic embryo transfer are promising, a potential increase in chromosomal abnormalities in the newborn is still concerning for the time being. Thus, clinical management of mosaic embryos depends on our ability to identify and interpret the factors that lead to aneuploid cells vanishing during development. Normal outcomes following the transfer of embryos deemed as mosaic lead us to consider several explanations, such as the technical overestimation of mosaicism and the possibility of some sort of “self-correction” during development. Even though signs of embryo plasticity are evident and aneuploid cells can be marginalized during early embryo differentiation [46], a self-correction mechanism to eliminate the aneuploid cells in human preimplantation embryos remains unproven [32]. In contrast to the animal model, in which a self-correcting mechanism to eliminate aneuploid cells through apoptosis and severe proliferative reduction has been demonstrated [36], convincing evidence in human embryos is still lacking. Even so, data from humans seem to support these findings, with preferential apoptosis in the ICM lineage in aneuploid–euploid mosaics [39] and increased cell division that potentially compensates for the slower proliferation or programmed death of aneuploid cells [40,41]. This represents the most promising model of normalization to a euploid constitution, and the presence of cfDNA, mRNA of apoptotic genes, and extracellular vesicles in BF, lends more support [49]. While the trisomic/monosomic model represents a potential method to overcome aneuploidy, the extremely low prevalence of UPD reported in human embryos [52] suggests that, even if conceivable, it is far from being the predominant mechanism of self-correction and could hardly be responsible for most of the aneuploidy correction. On the other hand, the healthy live births reported following the transfer of mosaic embryos may be due to an overestimation of chromosomal mosaicism in clinical practice. Thus, viable embryos would be classified as mosaic aneuploid because of either sampling bias from a simple TE biopsy or the technical limitations of the clinical methods currently used. The level of mosaicism is likely to depend on when the segregation error occurred and the concordance between TE and ICM is reported to be strictly related to this moment. Therefore, the ICM and TE karyotypes show high concordance in aneuploidy diagnosis, reflecting the meiotic segregation error, which is likely to be uniformly distributed across the embryo. In contrast, when mosaicism is diagnosed, the correlation with chromosomal content in the ICM was found to be different depending on the level of mosaicism [7]. Thus, the level of mosaicism seems to be a good predictor of the ICM karyotype, since low- to medium-level mosaicism (<50% aneuploid cells in a single TE sample) rarely affected the ICM and other regions, indicating a group of aneuploid cells highly confined to a restricted portion of the embryo. In contrast, when mosaicism impacted ≥50% of the TE sample (high-level mosaicism), aneuploidy displayed higher prevalence, and 65% of the embryos were actually associated with uniform aneuploidy across the embryo present in all the regions analyzed. Additionally, the common application of NGS as a testing methodology using copy number thresholds to identify the chromosomal content of the embryo has been reported to be poorly predictive in cases of mosaicism diagnosis. The assumption of “intermediate copy number profiles” as evidence of a mixture of euploid and aneuploid cells in a single biopsy may reflect not only a biological signal of mosaicism but also technical noise. Indeed, the technical noise distribution seems to differ between chromosomes, impacting most of the smaller ones [54]. Thus, artifactual intermediate copy numbers may arise from a suboptimal number of TE cells, undetected sample contamination, polyploidy, or the technology/algorithms used to amplify and normalize copy number data. The inaccuracy of the copy number strategy to predict mosaicism is reflected in 57% of embryos being deemed mosaic, which were reported to be truly euploid or fully aneuploid after rebiopsy [10]. Importantly, the mere use of a wider threshold range (80–20% instead of 70–30%) impacts the diagnostic accuracy, resulting in significantly higher false-positive mosaicism rates (79.5% versus 57.8%; *p* < 0.00001) [54]. Due to the negative impact that multiple rebiopsies would have on the outcome of a specific embryo, no correlation has been clearly found between initial TE diagnosis, the “real” chromosomal status obtained after evaluating multiple rebiopsies, and the clinical outcomes of each embryo. Even though direct evidence is still lacking, the percentage of euploidy (24.4% and 42.8%) and uniform aneuploidy (39.9% and 47.6%) reported in mosaic embryos after their reanalysis by Marin and collaborators [10] and Handyside and coworkers [57], respectively, are consistent with the reproductive potential and outcomes following mosaic embryo transfer [22]. These data, together with the differential distribution of aneuploid cells in the ICM depending on the level of mosaicism, found by Capalbo and co-workers [7], lead us to consider that when the transfer of low- to middle-level mosaics result in healthy outcomes, it may be because they were euploid but never actually mosaic (false positive) and, if present, the putative aneuploid cells, which originated from mitosis, would be strictly located in the TE. Conversely, a high-level mosaic could fail to implant simply because it would be uniformly aneuploid following a meiotic segregation error.

## 7. Future Perspectives

While blastocyst mosaicism represents a real phenomenon, its true impact and clinical relevance remain unclear. Data from several models indicated that the population of extraembryonic cells is particularly resilient to aneuploidy, whereby aneuploid cells tend to survive in the TE. ICM cells display significantly higher rates of apoptosis, suggesting the presence of an in vivo mechanism of self-correction. Further studies are required to unravel the cellular and molecular mechanisms underlying the reduction in aneuploid cells during early post-implantation development. In this context, it has been suggested that the TE may tolerate high rates of aneuploidy due to a lack of or a lax cell checkpoint regulation that would allow them to bypass the mitotic checkpoints, similar to pluripotent cells. Moreover, the upregulation of pro-apoptotic genes and a decline in apoptosis thresholds have been reported in mouse embryonic cells, in response to DNA damage at early post-implantation stages, but not in their extraembryonic counterparts. This could potentially be the target of forthcoming studies combined with future in vitro models aimed at uncovering the fate of aneuploid cells. Additionally, we must consider tissue-specific development that different chromosomal aneuploidies may potentially display, as it may determine the developmental competency of a concrete mosaic embryo. Setting the goal for future research strategies may imply renouncing embryonic developmental dogmas assuming, for instance, that, like cancer cells, the abnormal chromosomal content of extraembryonic tissue could entail physiological functions, as faster proliferation and invasiveness could even account for a developmental benefit. Regardless of whether mosaicism may be resolved during pregnancy or if aneuploid cells could be retained in the extraembryonic tissues, the poor concordance between TE diagnosis and the rest of the embryo calls into question the analytical and clinical validity of PGT-A. It seems evident that there is a lack of accuracy of mosaic PGT results (based on intermediate copy number thresholds) in predicting true mosaicism in post-implantation embryos or fetuses. Therefore, properly designed, large-scale, randomized clinical trials should be conducted to provide sufficient high-level evidence regarding the validity of PGT-A for identifying mosaicism and unraveling its reproductive potential. Until then, the current practice of diagnosing mosaicism using intermediate copy number thresholds should be limited to research settings unless benefits for IVF treatment are clearly established. It becomes mandatory to redesign current technologies and explore novel methods to improve the accuracy of mosaicism diagnosis, avoiding the misclassification of embryos that are, in fact, uniformly euploid or aneuploid. Several innovations may be considered, especially those able to distinguish the meiotic and mitotic origins of aneuploidy, since they can increase the specificity of mosaicism predictions. These strategies include meiomapping of the first and second polar body (PB) and genome-wide SNP genotyping/karyomapping of PB and TE samples. In combination with NGS-based copy number analysis of TE cells, this approach can target different haplotypes, providing a second analysis that can identify maternal or paternal backgrounds of trisomies and monosomies, and even detect polyploidy. In addition, as time-lapse parameters have been reported to be significantly different between mosaic and aneuploid embryos [74], morphokinetics and morphometrics [75] could potentially be explored as a complementary strategy to improve diagnostic specificity. Logically, specificity would be enhanced by minimizing the technical noise derived from genome amplification artefacts. This is the main asset of an emerging technology, an alternative single-cell WGA technology, locus-specific high-depth sequencing via primary template-directed amplification (PTA), which has proven to improve the reliability of amplification (the signal: noise ratio) by limiting the generation of amplicons to the original template. It uses exonuclease-resistant terminators in the reaction to limit the subsequent amplification from an exponential into a quasilinear process where the primary templates are mainly used and, thereby, potential error propagation from daughter amplicons is minimized. Additional approaches, such as low-depth sequencing of the direct library preparation (DLP), which permits the analysis of chromosome copy numbers but, most importantly, without preamplification, should be further explored [76]. Although this novel technology may represent an upcoming alternative to PGT, its validation requires further assessment. Upgrading the accuracy of diagnosis becomes mandatory to determine the proper value of reporting mosaicism in clinical practice, its medical relevance, and in establishing its putative role in decision-making.

## Figures and Tables

**Table 1:** Correlation between TE and ICM assessment.

	PGT-A	Cases		Correlation	
			Euploidy	Aneuploidy	Mosaicism
Fragouli et al., 2008 [64]	FISH-aCGH	10	100% (4/4)	100% (6/6)	-
Northrop et al., 2010 [51]	SNP array	21		42.8% (9/21)	
Capalbo et al., 2013 [65]	FISH aCGH	85	100% (20/20)	90.2% (46/51)	85.7% (12/14)
Huang et al., 2017 [66]	aCGH	51	-	84.3% (43/51)	-
Victor et al., 2019 [40]	NGS	93	-	96.8% (90/93)	-
Liu et al., 2012 [62]	aCGH	13	-	30.8% (4/13)	-
Tsuiko et al., 2018 [69]	NGS	14	100% (9/9)	100% (2/2)	75.0% (3/4)
Popovic et al., 2019 [35]	NGS	24	-	100% (8/8)	18.8% (3/16)
Chuang et al., 2018 [70]	NGS	29	100% (8/8)	43.8% (7/16)	20.0% (1/5)
Wu et al., 2021 [63]	NGS	91	-	-	27.5% (25/91)
Victor et al., 2019 [55]	NGS	8	-	100% (3/3)	40.0% (2/5)
					Euploid 60.0% (3/5)
Lawrenz et al., 2019 [71]	NGS	84	93.2% (41/44)	92.5% (37/40)	-
Sachdev et al., 2020 [72]	NGS	32	99.5%	97.3%	35.2%
Lin et al., 2020 [19]	NGS	14			50.0% (7/14)
Low-range mosaic					Euploid 50.0% (7/14)
					Aneuploid 0% (0/24)
High-range mosaic	NGS	27			37.0 (10/27)
					Euploid 40.7% (11/27)
					Aneuploid 22.0% (6/27)
Capalbo et al., 2021 [7]	NGS				
Low-range mosaic (20–30%)		37	-	-	0% (1/148)
					Euploid 100% (147/148)
Medium-range mosaic (30–50%)		31	-	-	4.4% (2/46)
					Euploid 93.4% (43/46)
					Aneuploid 2.2% (1/46)
High-range mosaic (50–70%)		5	-	-	20.0% (4/20)
					Euploid 15.0% (3/20)
					Aneuploid 65.0% (13/20)
Chavli et al., 2022 [73]	NGS				
Low-range mosaic		3			0% (0/3)
					Euploid 100% (3/3)
High-range mosaic		4			25.0% (1/4)
					Euploid 25.0% (1/4)
					Aneuploid 50.0% (2/4)

## Data Availability

All data are incorporated into the article.
